# Food Safety Knowledge, Attitudes, and Practices Among Dormitory Students at the University of Sharjah: A Cross-Sectional Study

**DOI:** 10.7759/cureus.112085

**Published:** 2026-07-05

**Authors:** Aysha M Hussein, Hana A Elbery, Rama W Rabboua, Rama A Quran, Yahia R Elgharib, Ali A Alkhalidi, Abdulrahman A Aljneibi, Amal Hussein, Wafa K Alnakhi

**Affiliations:** 1 College of Medicine, University of Sharjah, Sharjah, ARE; 2 Department of Family and Community Medicine and Behavioral Sciences, University of Sharjah, Sharjah, ARE

**Keywords:** attitudes, dormitory students, foodborne illnesses, food handling, food safety, knowledge, practices

## Abstract

Background

Foodborne illnesses remain a major global public health concern, often linked to poor hygiene and unsafe food handling. Dormitory students are particularly vulnerable due to shared kitchen facilities and varying levels of food safety awareness. This study aims to assess knowledge, attitudes, and practices (KAPs) regarding food safety among dormitory students at the University of Sharjah.

Materials and methods

A cross-sectional study was conducted at the University of Sharjah during 2024-2025. The sample was selected using convenience sampling, targeting at least 385 students. A self-administered questionnaire adapted from validated studies was distributed among university dormitories. It included demographic data, multiple-choice questions (MCQs) assessing knowledge and practices, and a Likert-scale section assessing attitudes toward food safety. Data were analyzed using SPSS version 29. The chi-square test was used to assess the association between categorical variables, with statistical significance set at p < 0.05.

Results

A total of 399 dormitory students participated. Of these, 249 (62.4%) were female. Most students were aged 20 years or older. A total of 238 (59.6%) were enrolled in medical-related majors. The sample included students from women’s dormitories (150, 37.6%), men’s dormitories (150, 37.6%), and medical/nursing dormitories (99, 24.8%). Age and gender were significantly associated with practice level (p = 0.048 and p < 0.001, respectively), with students aged ≥20 years and female students demonstrating better practices. More than half of the medicine and health sciences students had good knowledge (130, 54.6%) compared with non-medical students (p = 0.004). Students in the medical/nursing dormitory reported higher levels of good knowledge (63, 63.6%) and good practice (55, 55.6%) (p = 0.001 and p < 0.001, respectively). Higher knowledge was associated with better attitudes and better practices, and a higher attitude level was associated with a better practice level.

Conclusions

Dormitory students at the University of Sharjah demonstrated poor attitudes and moderate levels of food safety knowledge and practices. The observed knowledge-attitude gap represents a public health concern. Universities should implement targeted educational interventions to improve food safety awareness and promote the translation of knowledge into appropriate attitudes and practices.

## Introduction

Foodborne illness refers to any disease resulting from the consumption of contaminated food or beverages, typically caused by bacteria, viruses, parasites, or chemical substances [1]. It remains a significant global public health concern, with approximately one in every ten people worldwide falling ill each year after consuming unsafe food, leading to an estimated 420,000 deaths annually [2]. This substantial burden reflects ongoing challenges related to inadequate hygiene practices and unsafe food handling behaviors at both household and community levels. In the United Arab Emirates, foodborne diseases continue to pose a public health issue, with 2,081 reported cases of food poisoning in 2022, highlighting the local relevance of this problem [3]. Several studies conducted in the UAE have examined food safety knowledge and practices among different populations. For example, a study conducted in Dubai in 2022 assessed knowledge, practices, and risk perception related to foodborne illnesses among females living in Dubai and found that, although participants demonstrated acceptable knowledge in some areas, gaps were identified in safe food handling and storage practices [4]. More recently, a cross-sectional study conducted in the United Arab Emirates in 2024 evaluated food safety knowledge and attitudes among adults and reported that only about 46% of participants demonstrated adequate food safety knowledge, despite generally positive attitudes toward food safety [5]. These findings suggest that although awareness of food safety exists in the UAE, gaps in knowledge and practices remain, indicating the need for continued food safety education and public health interventions. University dormitory students represent a particularly vulnerable group due to shared kitchen facilities, time constraints related to academic demands, and reliance on quick or improperly stored meals. In addition, the cultural diversity within the student population may influence food handling behaviors and perceptions of food safety. From a public health and behavior change perspective, it is important to recognize that knowledge alone does not necessarily lead to safe practices and that attitudes and environmental factors play a significant role in shaping behavior.

Despite these risks, research specifically examining food safety knowledge, attitudes, and practices (KAPs) among dormitory students in the UAE remains limited. Most existing studies have focused on the general population or food handlers, with little attention given to students living in shared residential settings. Addressing this gap is important to inform targeted health promotion strategies and behavior-based interventions aimed at reducing the risk of foodborne illness in this population. Therefore, this study aims to evaluate the levels of KAPs regarding food safety among dormitory students at the University of Sharjah and to examine their associations with gender, academic major, and dormitory type.

## Materials and methods

Study design and inclusion criteria

A descriptive cross-sectional study was conducted at the University of Sharjah, United Arab Emirates (UAE), between February 2024 and November 2025, with data collection conducted specifically between March and April 2025. The target population included male and female students aged 18-24 years residing in the University of Sharjah medical/nursing, women’s, or men’s dormitories during the data collection period, regardless of nationality, socioeconomic status, or academic major. Participants who met the inclusion criteria, students aged 18-24 years living in the University of Sharjah dormitories and willing to participate with informed consent, were included in the analysis, provided that they fully completed the questionnaire. Students who did not meet these eligibility criteria were excluded, including those with missing data, those unable to understand Arabic or English, and students from dormitories of universities other than the University of Sharjah.

Sample size

The required sample size was calculated using Cochran’s formula for cross-sectional studies: (n = Z^2^p(1-p)/SE^2^), where (Z = 1.96) for a 95% confidence level. The expected prevalence was set at 50% (0.5) due to the lack of previous evidence reporting adequate knowledge of food safety among dormitory students. With a margin of error of 5% (0.05), the minimum required sample size was 385 participants, who were recruited using convenience sampling.

Study tool

Data were collected using a self-administered questionnaire adapted and modified from previously published studies [5-7]. Modifications included tailoring questions to ensure relevance to the target population and rewording items for clarity. The survey was initially developed in English and then translated into Arabic to reach a broader audience. However, no formal validation procedures, such as forward-backward translation, pilot testing of the translated version, or reliability analysis, were performed. The questionnaire consisted of four sections with a total of 40 items and required approximately 18 minutes to complete. The first section included eight questions on sociodemographic characteristics, including age, gender, nationality, year of study, major, dormitory, history of food poisoning, and whether participants usually cooked their own food. The second section assessed knowledge of safe food handling through 13 items. The third section evaluated attitudes toward food safety through seven items. The fourth section assessed food handling practices through 12 items.

Data collection

The questionnaire was distributed via Microsoft Forms. Participants were recruited online through WhatsApp groups, in person using QR codes in dormitories, and through word of mouth among students. Before starting the questionnaire, participants were provided with an information sheet explaining the study purpose and procedure. They were informed that participation was voluntary, responses were anonymous to ensure confidentiality, and they could withdraw at any time without consequences. The response rate was 91%, calculated as the proportion of completed questionnaires among eligible students.

Scoring methods

For the knowledge and practice sections, each correct response was assigned one point, while incorrect responses were assigned zero points. For the attitude section, a five-point Likert scale (“strongly agree,” “agree,” “neutral,” “disagree,” and “strongly disagree”) was used. Responses reflecting a positive attitude toward food safety were assigned one point, while negative responses were assigned zero points. Likert-scale responses were combined into two categories: “strongly agree” and “agree” were grouped as positive responses, while “neutral,” “disagree,” and “strongly disagree” were grouped as negative responses. Total scores for knowledge, attitude, and practice were calculated by summing item scores. The Kolmogorov-Smirnov test indicated that all scores were not normally distributed; therefore, medians were used as cut-off values. Participants scoring above the median were classified as having good knowledge, attitude, or practice, while those scoring at or below the median were classified as poor. The maximum knowledge score was 17, as some items had multiple correct answers. A score above 10 indicated good knowledge, while scores of 10 or less indicated poor knowledge. The maximum attitude score was six, as one item was not scored; scores above three indicated a positive attitude, while scores of three or less indicated a negative attitude. The maximum practice score was 12; scores above six indicated good practice, while scores of six or less indicated poor practice.

Statistical analysis

Statistical analysis was performed using SPSS version 29.0. Descriptive statistics, including frequencies, percentages, medians, and IQRs, were used to summarize the data. Normality was assessed using the Kolmogorov-Smirnov test. The chi-square test was used for bivariate analysis to examine associations between sociodemographic characteristics and knowledge, attitude, and practice levels. A p-value < 0.05 was considered statistically significant.

Ethical considerations

Ethical approval was obtained from the Ethics and Research Committee (ERC) of the Medical College at the University of Sharjah, under reference number REC-25-03-16-18-S. Informed consent was obtained from all participants before participation.

## Results

A total of 399 dormitory students participated in the study, of whom 249 (62.4%) were female. The mean age was 20 years. Most participants were from Middle Eastern countries, 299 (74.9%), followed by Asian, 59 (14.8%), and African, 39 (9.8%), nationalities. The majority of students were enrolled in medical-related majors, 238 (59.6%), while 161 (40.4%) were in non-medical majors. Dormitory students were mainly second-year students, 140 (35.1%), followed by first-year, 96 (24.1%), and third-year students, 59 (14.8%). The sample included students from the women’s dormitory and men’s dormitory, 150 each (37.6% each), and medical/nursing dormitories, 99 (24.8%) (Table 1).

**Table 1:** Sociodemographic characteristics of the study participants.

Variable	Options	Frequency	%
Age	<20 years	169	42.4
	≥20 years	230	57.6
Gender	Male	150	37.6
	Female	249	62.4
Ethnicity	Middle Eastern	299	74.9
	Asian	59	14.8
	African	39	9.8
	North/South American	2	0.5
Major	Medicine and health sciences	238	59.6
	Non-medical-related majors	161	40.4
Academic year	Foundation year	35	8.8
	Year 1	96	24.1
	Year 2	140	35.1
	Year 3	59	14.8
	Year 4	46	11.5
	Year 5+	23	5.8
Dormitory	Women’s dormitories	150	37.6
	Men’s dormitories	150	37.6
	Medical/nursing dormitories	99	24.8

Regarding cooking frequency in dormitories, 162 (40.6%) students reported sometimes cooking in the dormitory, followed by those who usually ordered food or stored food, 92 (23.1%), and those who rarely cooked, 90 (22.6%). The main sources of food safety information were family and friends, 155 (38.8%), followed by social media/internet, 154 (38.6%), and healthcare professionals, 36 (9%).

Overall, students demonstrated moderate knowledge levels, with 194 (48.6%) classified as having good knowledge and 205 (51.4%) as having poor knowledge. The majority of students demonstrated a poor overall attitude level, 302 (75.7%), while 97 (24.3%) demonstrated a good attitude. Practice levels were approximately balanced, with 193 (48.4%) showing good practice and 206 (51.6%) showing poor practice. Table 2 presents the full distribution of knowledge, attitude, and practice items and corresponding scores.

**Table 2:** Food safety knowledge, attitude, and practice questions. ¹ The remaining 134 (33.6%) participants reported that they did not use the shared dormitory air fryer. ² The remaining 140 (35.1%) participants selected “not applicable” for this item. ³ The remaining 169 (42.4%) participants selected “not applicable” for this item.

Section	Correct response n (%)	Incorrect response n (%)
Knowledge questions		
True or false: Using the same sink for washing dishes and hands increases the risk of food contamination. (True)	300 (75.2)	99 (24.8)
True or false: Long and/or painted nails increase the risk of food contamination. (True)	383 (96.0)	16 (4.0)
True or false: Washing hands after handling raw food reduces the transmission of food-related germs. (True)	378 (94.7)	21 (5.3)
True or false: Using the same cutting board for raw meat and vegetables without cleaning it first can cause cross-contamination. (True)	373 (93.5)	26 (6.5)
For how long is it sufficient to wash hands? (20 seconds)	62 (15.5)	337 (84.5)
What is the correct way to wash vegetables? (Cold running water)	77 (19.3)	322 (80.7)
What is the maximum refrigerator temperature required to preserve food safety? (4°C)	134 (33.6)	265 (66.4)
Which of the following can cause food poisoning when eaten? (Raw or undercooked eggs and meat)	320 (80.2)	79 (19.8)
Which is the most important factor for preventing food poisoning? (Washing hands properly after eating)	139 (34.8)	260 (65.2)
Signs of spoiled canned food: Bulging can or lid or a broken seal. (Yes)	46 (60.5)	30 (39.5)
Signs of spoiled canned food: A can or lid that shows signs of corrosion. (Yes)	41 (53.9)	35 (46.1)
Signs of spoiled canned food: Food that has oozed or seeped under the jar’s lid. (Yes)	44 (57.9)	32 (42.1)
Signs of spoiled canned food: Gassiness, or bubbles in the jar/can. (Yes)	36 (47.4)	40 (52.6)
Signs of spoiled canned food: Food appearing cloudy, mushy, or moldy. (Yes)	48 (63.2)	28 (36.8)
What is the best way to check that chicken is sufficiently cooked? (Thermometer reading)	147 (36.8)	252 (63.2)
Which food safety practice reduces the risk of contamination the most? (Cooking meat to the appropriate temperature)	266 (66.7)	133 (33.3)
Attitude questions	Positive attitude n (%)	Negative attitude n (%)
Food safety is more important than taste. (Strongly agree/agree)	351 (88.0)	48 (12.0)
A person having diarrhea, flu, or a sore throat should not prepare food for others. (Strongly agree/agree)	343 (85.9)	57 (14.1)
Using hand sanitizers can completely replace hand washing during food preparation and handling. (Neutral/disagree/strongly disagree)	183 (45.9)	216 (54.1)
Food that has been left outside for a long time should be thrown away immediately. (Strongly agree/agree)	315 (78.9)	84 (21.1)
Food should only be thrown away once it shows visible signs of spoilage. (Neutral/disagree/strongly disagree)	142 (35.6)	257 (64.4)
Kitchen thermometers are not necessary for measuring the doneness of meat. (Neutral/disagree/strongly disagree)	172 (43.1)	227 (56.9)
Is there a need for targeted awareness and educational programs to improve food safety knowledge and practices among dormitory students? (Strongly agree/agree)	323 (81.0)	76 (19.0)
Practice questions	Correct response n (%)	Incorrect response n (%)
A refrigerator has three shelves; on which shelf should raw meat be placed? (Bottom shelf)	133 (33.3)	266 (66.7)
After cutting raw meat, what should be done before using the knife again? (Wash)	263 (65.9)	136 (34.1)
How long should leftover foods be heated? (Until boiling hot)	80 (20.1)	319 (79.9)
Taking jewelry off when preparing food. (Yes)	228 (57.1)	171 (42.9)
If you or your roommate will be several hours late for a hot meal, where should the meal be kept? (Refrigerator and reheat)	188 (47.1)	211 (52.9)
How do you wash your hands before preparing food or eating? (Soap and cold water)	325 (81.5)	74 (18.5)
Do you clean the shared dormitory air fryer before or after personal use? (Yes)^1^	140 (35.1)	125 (31.3)
For those using a cutting board, do you use the same cutting board for different food items you consume, such as chicken, meat, fruits, and vegetables? (No)^2^	125 (31.3)	134 (33.6)
If you use the same cutting board, do you clean it between different food items? (Yes)^3^	178 (44.6)	52 (13.1)
What do you use to clean the kitchen counters? (Disinfectant cleaner/soap and water)	345 (86.5)	54 (13.5)
What do you do if you see food improperly stored/prepared in the dormitory kitchen? (Report/correct)	274 (68.7)	125 (31.3)
What should be done if you find expired food in the dormitory kitchen? (Dispose of it)	272 (68.2)	127 (31.8)

For knowledge, the median score was 10 (IQR of 4). Significant associations were found with dormitory type and major. Students from medical/nursing dormitories and those in medical and health sciences majors demonstrated higher knowledge levels, with good knowledge reported in 63 (63.6%) (p = 0.001) and 130 (54.6%) (p = 0.004), respectively (Table 3).

**Table 3:** Association between knowledge level and sociodemographic characteristics. *Statistically significant at p < 0.05. P-values were calculated using the chi-square test.

Demographics	Options	Poor knowledge n (%)	Good knowledge n (%)	Chi-square value	p-value
Age	<20 years	86 (50.9)	83 (49.1)	0.028	0.866
	≥20 years	119 (51.7)	111 (48.3)	-	-
Gender	Male	79 (52.7)	71 (47.3)	0.16	0.689
	Female	126 (50.6)	123 (49.4)	-	-
Ethnicity	Middle Eastern	146 (48.8)	153 (51.2)	7.572	0.056
	Asian	40 (67.8)	19 (32.2)	-	-
	African	18 (46.2)	21 (53.8)	-	-
	North/South American	1 (50.0)	1 (50.0)	-	-
Major	Medicine and health sciences	108 (45.4)	130 (54.6)	8.501	0.004*
	Non-medical-related majors	97 (60.2)	64 (39.8)	-	-
Academic year	Foundation year	14 (40.0)	21 (60.0)	8.307	0.14
	Year 1	55 (57.3)	41 (42.7)	-	-
	Year 2	72 (51.4)	68 (48.6)	-	-
	Year 3	26 (44.1)	33 (55.9)	-	-
	Year 4	29 (63.0)	17 (37.0)	-	-
	Year 5+	9 (39.1)	14 (60.9)	-	-
Dormitory	Women’s dormitories	90 (60.0)	60 (40.0)	13.497	0.001*
	Men’s dormitories	79 (52.7)	71 (47.3)	-	-
	Medical/nursing dormitories	36 (36.4)	63 (63.6)	-	-

For attitude levels, the median score was 3 (IQR of 0). Significant associations were found with dormitory type and major. Students from medical/nursing dormitories and medical and health sciences majors demonstrated better attitudes, with good attitude levels of 44 (44.4%) (p < 0.001) and 70 (29.4%) (p = 0.004), respectively (Table 4). In addition, 323 (80.9%) students supported the implementation of targeted awareness and educational programs to improve food safety knowledge and practices among dormitory students.

**Table 4:** Association between attitude level and sociodemographic characteristics. *Statistically significant at p < 0.05. P-values were calculated using the chi-square test.

Demographics	Options	Poor attitude n (%)	Good attitude n (%)	Chi-square value	p-value
Age	<20 years	135 (79.9)	34 (20.1)	2.8	0.094
	≥20 years	167 (72.6)	63 (27.4)	-	-
Gender	Male	121 (80.7)	29 (19.3)	3.236	0.072
	Female	181 (72.7)	68 (27.3)	-	-
Ethnicity	Middle Eastern	223 (74.6)	76 (25.4)	1.38	0.71
	Asian	47 (79.7)	12 (20.3)	-	-
	African	30 (76.9)	9 (23.1)	-	-
	North/South American	2 (100.0)	0 (0.0)	-	-
Major	Medicine and health sciences	168 (70.6)	70 (29.4)	8.341	0.004*
	Non-medical-related majors	134 (83.2)	27 (16.8)	-	-
Academic year	Foundation year	30 (85.7)	5 (14.3)	7.344	0.196
	Year 1	79 (82.3)	17 (17.7)	-	-
	Year 2	101 (72.1)	39 (27.9)	-	-
	Year 3	45 (76.3)	14 (23.7)	-	-
	Year 4	31 (67.4)	15 (32.6)	-	-
	Year 5+	16 (69.6)	7 (30.4)	-	-
Dormitory	Women’s dormitories	126 (84.0)	24 (16.0)	29.46	p < 0.001*
	Men’s dormitories	121 (80.7)	29 (19.3)	-	-
	Medical/nursing dormitories	55 (55.6)	44 (44.4)	-	-

For practice levels, the median score was 6 (IQR of 3). Significant associations were found with age, gender, and dormitory type. Students aged ≥20 years, female students, and those living in the women’s dormitories demonstrated better practices, with good practice levels of 121 (52.6%) (p = 0.048), 143 (57.4%) (p < 0.001), and 88 (58.7%) (p < 0.001), respectively (Table 5).

**Table 5:** Association between practice level and sociodemographic characteristics. *Statistically significant at p < 0.05. P-values were calculated using the chi-square test.

Demographics	Options	Poor practice n (%)	Good practice n (%)	Chi-square value	p-value
Age	<20 years	97 (57.4)	72 (42.6)	3.905	0.048*
	≥20 years	109 (47.4)	121 (52.6)	-	-
Gender	Male	100 (66.7)	50 (33.3)	21.764	p < 0.001*
	Female	106 (42.6)	143 (57.4)	-	-
Ethnicity	Middle Eastern	152 (50.8)	147 (49.2)	2.519	0.472
	Asian	33 (55.9)	26 (44.1)	-	-
	African	19 (48.7)	20 (51.3)	-	-
	North/South American	2 (100.0)	0 (0.0)	-	-
Major	Medicine and health sciences	132 (55.5)	106 (44.5)	3.47	0.062
	Non-medical-related majors	74 (46.0)	87 (54.0)	-	-
Academic year	Foundation year	22 (62.9)	13 (37.1)	6.941	0.225
	Year 1	50 (52.1)	46 (47.9)	-	-
	Year 2	78 (55.7)	62 (44.3)	-	-
	Year 3	26 (44.1)	33 (55.9)	-	-
	Year 4	18 (39.1)	28 (60.9)	-	-
	Year 5+	12 (52.2)	11 (47.8)	-	-
Dormitory	Women’s dormitories	62 (41.3)	88 (58.7)	21.995	p < 0.001*
	Men’s dormitories	100 (66.7)	50 (33.3)	-	-
	Medical/nursing dormitories	44 (44.4)	55 (55.6)	-	-

Associations were assessed between knowledge and attitude, knowledge and practice, and attitude and practice. Significant associations were observed in all pairs. Higher knowledge was associated with better attitudes and better practices, and better attitudes were associated with better practices (Table 6).

**Table 6:** Associations between knowledge, attitude, and practice levels. *Statistically significant at p < 0.05. P-values were calculated using the chi-square test.

Comparison	Level	Poor practice n (%)	Good practice n (%)	Chi-square value	p-value
Knowledge level vs. practice level	Poor knowledge	119 (58.0)	86 (42.0)	-	-
	Good knowledge	87 (44.8)	107 (55.2)	6.958	0.008*
Comparison	Level	Poor attitude n (%)	Good attitude n (%)	Chi-square value	p-value
Knowledge level vs. attitude level	Poor knowledge	178 (86.8)	27 (13.2)	-	-
	Good knowledge	124 (63.9)	70 (36.1)	28.436	p < 0.001*
Comparison	Level	Poor practice n (%)	Good practice n (%)	Chi-square value	p-value
Attitude level vs. practice level	Poor attitude	168 (55.6)	134 (44.4)	-	-
	Good attitude	38 (39.2)	59 (60.8)	7.959	0.005*

## Discussion

This study demonstrated that dormitory students at the University of Sharjah exhibited moderate levels of food safety knowledge and practices, alongside predominantly poor attitudes. Similar patterns have been reported in previous studies among university populations, where knowledge is generally moderate to good, while attitudes tend to be less favorable and less consistent [8-10].

These findings indicate that although overall knowledge and practice levels were moderate, attitudes were predominantly poor. Students with better knowledge were more likely to demonstrate positive attitudes and good food safety practices. Similar relationships among KAP domains have been reported among university students in Bangladesh, Jordan, and China, where higher food safety knowledge was associated with improved attitudes and safer food handling behaviors [6-9]. By focusing on dormitory residents in the United Arab Emirates, this study contributes context-specific evidence from a population potentially at increased risk due to communal living conditions and shared kitchen environments.

Gender, age, and major emerged as key determinants of food safety practices. Female students, students aged 20 years and above, and those residing in women’s dormitories reported better food safety practices. The finding regarding female students is consistent with previous research indicating greater adherence to hygiene-related behaviors among females [8,10-12]. Students enrolled in medical and health-related majors demonstrated higher knowledge levels, likely reflecting greater curricular exposure to health sciences content, as supported by earlier studies [8,9,11].

Low correct response rates were observed for questions assessing knowledge regarding handwashing duration, correct refrigerator temperature, washing vegetables, reheating leftovers, and appropriate food storage practices, consistent with previously published studies [6,7,10].

The findings from this study are consistent with intervention-based literature highlighting the importance of experiential and structured education in improving food safety outcomes. Evidence suggests that practical, skills-based training approaches are effective in improving both knowledge retention and behavioral change [13,14]. A longitudinal study among Ontario high school students demonstrated improvements in food safety knowledge and attitudes following a food handler training program, further supporting the value of structured educational interventions in promoting positive food safety behaviors [15].

From a public health perspective, these findings emphasize the need for integrated interventions targeting both behavioral and environmental determinants. Universities should prioritize targeted education and awareness programs with practical food safety training, particularly for non-health science students, to bridge the gap between knowledge and attitude. In addition, institutional strategies such as routine kitchen inspections, enforcement of hygiene standards, and provision of adequate food storage and cleaning facilities may reduce structural barriers to safe behavior. Behavioral reinforcement strategies, including reminders and peer-led initiatives, may further enhance compliance with safe food handling practices.

This study has several limitations. The use of convenience sampling may limit the generalizability of the findings, as students with greater interest in health and food safety topics may have been more likely to participate, potentially leading to an overestimation of knowledge, attitudes, and food safety practices within the study population. Furthermore, the use of self-reported data may have introduced recall and social desirability bias. The cross-sectional design limits causal inference, and findings from a single institution may not be representative of other universities. Although the study achieved an adequate sample size and included multiple dormitory types (women’s, men’s, and medical/nursing dormitories), broader multi-institutional studies are required to improve external validity. The absence of psychometric validation may affect the interpretation of the knowledge, attitude, and practice scores. While preliminary adaptation of the instrument was conducted, further psychometric testing is recommended to strengthen the reliability and validity of the study tool.

Despite these limitations, this study provides novel insight into food safety KAPs among dormitory students in the United Arab Emirates. The findings establish a baseline for future interventions and highlight the need for integrated educational and environmental strategies to improve food safety behaviors in university residential settings.

## Conclusions

Overall, the findings show significant gaps in students’ attitudes despite moderate levels of knowledge and practices. The differences observed based on gender, academic background, and dormitory type highlight the influence of educational exposure and living environment on food safety practices. The significant association between higher knowledge and improved attitudes and practices emphasizes the importance of strengthening students’ food safety awareness. Universities should implement targeted educational and awareness programs to support proper food safety practices and promote the translation of knowledge into healthy daily behaviors. The observed knowledge-attitude gap represents an important public health concern, as students with adequate food safety knowledge may still demonstrate poor attitudes toward food safety, thereby increasing the risk of foodborne illnesses, disease transmission in shared residential settings, and associated academic and healthcare burdens.

The study has several limitations. Convenience sampling limits representativeness, and the cross-sectional design precludes causal inference. In addition, findings from a single institution may not be generalizable to other universities. Future studies should expand the scope to include different campuses within the University of Sharjah, such as Khorfakkan, Kalba, and Al Dhaid, or incorporate multiple universities with diverse dormitory settings. Future research should also consider longitudinal and intervention-based designs to assess changes in food safety knowledge and practices over time, similar to studies conducted among high school students in Ontario following food handler training programs. Furthermore, comprehensive psychometric validation is recommended to ensure the reliability and validity of the questionnaire, including assessment of multiple validity domains.
